# Prevalence of *Salmonella* species and factors associated with contamination of mechanically recovered poultry meat imported into South Africa, 2016–2017

**DOI:** 10.14202/vetworld.2023.2236-2243

**Published:** 2023-11-11

**Authors:** Tandile Nwabisa Ndobeni, Kudakwashe Magwedere, Daniel Nenene Qekwana

**Affiliations:** 1 Section of Veterinary Public Health, Department of Paraclinical Sciences, Faculty of Veterinary Science, University of Pretoria, South Africa; 2 Directorate of Veterinary Public Health, Department of Land Reform and Rural Development, South Africa

**Keywords:** foodborne, import, mechanically recovered poultry meat, risk factors, *Salmonella*, zoonoses

## Abstract

**Background and Aim:**

Mechanically recovered meat (MRM) products have been linked to outbreaks of human salmonellosis. However, no studies have investigated the prevalence of *Salmonella* species in MRM products in South Africa despite the products being imported. Therefore, this study aimed to estimate the prevalence and factors associated with *Salmonella* spp. contamination of mechanically recovered poultry meat (MRPM) imported into South Africa.

**Materials and Methods:**

This study used secondary data of MRPM consignments imported through a port entry into South Africa from May 2016 to December 2017. Crude and factor-specific proportions of *Salmonella* positive MRPM and their 95% confidence intervals were calculated. A logistic regression model was used to assess the association among country, season, importer, year, and MRPM *Salmonella* status.

**Results:**

A total of 8127 MRPM consignments were imported. Seventeen percentages (17.18%, 985/5733) of consignments tested positive for *Salmonella* species and only 364 isolates were serotyped. *Salmonella* Heidelberg (73.90%, 269/364) was the most common serotype followed by *Salmonella* Infantis (6.59%, 24/364), *Salmonella* Salamae (4.67%, 17/364), and *Salmonella* Schwarzengrund (3.57%, 13/364). The odds of a consignment testing positive for *Salmonella* spp. was higher among consignments from country-B (Odds Ratio [OR]: 3.958, p < 0.0001) compared to “All others.” The odds of testing positive for *Salmonella* were also higher among consignments imported in autumn (OR: 1.488, p < 0.0001) but lower among those imported in spring (OR: 0.767, p = 0.0004) and summer (OR: 0.843, p < 0.0001) when compared to the winter season. Consignments imported in 2016 compared to 2017 were 1.563 times (p < 0.0001) as likely to test positive for a *Salmonella* species.

**Conclusion:**

*Salmonella* species were reported in MRPM consignments in this study with *Salmonella* Heidelberg being the most common serotype. Furthermore, some *Salmonella* serotypes reported in this study have been implicated in foodborne disease outbreaks. Country of origin, season, and year of importation were significantly associated with the odds of a consignment testing positive for *Salmonella* species.

## Introduction

Mechanically recovered meat (MRM) is derived by separating meat from bone [1–3]. It is used to produce a variety of products, including mince, chunks, and soup packs. Most of the MRM in South Africa is imported [4]. In addition, the increased consumption of poultry meat in South Africa [5] has made mechanically recovered poultry meat (MRPM) an inexpensive alternative source of protein [1, 2]. Despite the high consumption of MRPM, studies investigating its role as a source of salmonellosis in South Africa are limited. This is concerning as *Salmonella* species have been reported as contaminants in poultry and poultry products in South Africa [6–9]. *Salmonella* is a facultative, anaerobic, rod-shaped, and Gram-negative bacterium belonging to either the *Salmonella bongori* or *Salmonella enterica* species group [10]. Most *Salmonella* species have a broad spectrum of hosts, with a few being restricted to one specific host [11]. Humans and animals are asymptomatic carriers of the bacterium; however, clinical conditions have been reported in both species [12]. Several *Salmonella* spp. have been linked to human foodborne illnesses [13], mostly due to the consumption of poultry products [5, 14–18]. Contact with a contaminated environment has also been reported as another route of *Salmonella* infection in humans [12, 18–22].

Although salmonellosis is usually self-limiting, clinical signs, including diarrhea, nausea, vomiting, and abdominal pain [10, 23] have been reported especially among individuals with underlying clinical conditions and immunocompromised [24]. Mortality rates of up to 7.1% in children and up to 15.6% in adults have been reported [25, 26].The high mortality has been attributed to high levels of antimicrobial resistance which impacts patient care and prognosis [27, 28]. In addition, the disease has been associated with increased health costs and loss of human productivity [29, 30]. Of concern is the increasing prevalence of multidrug resistant *Salmonella* species associated with higher morbidity in humans [28, 31], including resistance to amphenicols, sulphonamides, carbapenems, macrolides, and tetracycline [28, 32, 33]. Studies attribute this to previous exposure to antimicrobials and a history of international travel [27, 31, 34, 35]. For example, international travel has been identified as a risk factor for acquiring extended-spectrum beta-lactamases-producing *Enterobacteriaceae* [36, 37]. Similarly, there is evidence of *Salmonella* infections associated with international travel [38].

This study aimed to estimate the prevalence of *Salmonella* species in MRPM imported into South Africa and identify factors associated with importing a *Salmonella* spp. contaminated consignment. The information generated from this study will help guide animal and public health policies on controlling and preventing *Salmonella* outbreaks in South Africa. Furthermore, this study can be used as a baseline for developing a quantitative risk assessment framework for importing MRPM in South Africa.

## Materials and Methods

### Ethical approval

This study was approved by the Department of Agriculture, Land Reform and Rural Development (DALRRD), and the Animal Ethics and Research Ethics Committees of the University of Pretoria (REC 182-19).

### Study period and location

The study was conducted from October 2019 to May 2021 in Pretoria, South Africa, using laboratory records received from DALRRD of *Salmonella* results of MRPM imported into South Africa from May 2016 to December 2017.

### Study setting

The South African government assesses the risk of importing a commodity into the country based on among others the disease status of the exporting country, answers to a questionnaire, inspection visits, disease control, and surveillance programs [39]. Once the risk is deemed to be negligible in line with the Animal Diseases Act, 1984 (Act No. 35 of 1984) and the Meat Safety Act, 2000 (Act No. 40 of 2000), the country is approved, and specific establishments are listed as eligible for export of MRPM to South Africa.

All importers are required to apply for an import permit from the DALRRD. The imported consignment is accompanied by the veterinary health certificate issued by the veterinary authority of the exporting country. A Veterinary Import Permit is to be used only once and it is valid for 6 months. On arrival at the port of entry in South Africa, each consignment of the MRPM undergoes documentary inspection, and physical inspection followed by a determination of the level of microbiological contamination of the product to assess its risk to human or animal populations in South Africa [39].

Under the Meat Safety Act, 2000 (Act No.40 of 2000), microbiological testing is mandatory for compliance monitoring. On arrival and during cold storage, five samples from selected consignment packages are randomly collected and placed in labeled sterile sample bags for further laboratory analysis. In the laboratory, the following method of isolation and characterization was followed as described by Gelaw *et al*. [40] and Carroll *et al*. [41]. Briefly, 25 g of MRPM was incubated for 18–24 h at 37°C and another 18–24 h at 42°C in a Rappaport Vassiliadis (Oxoid^®^, Basingstoke, England) enrichment broth. Subcultures from enrichment media were grown on xylose-lysine deoxycholate agar (Difco^®^, Lansing, USA) selective solid media and incubated at 37°C for 18–24 h. Suspected *Salmonella* isolates were then validated by performing biochemical tests on black colonies with a pink border. In addition, confirmed *Salmonella* isolates were serotyped using a battery of somatic O and flagellar H polyvalent and monovalent antisera following the Kauffmann-White classification system. The results of the bacteriological tests are captured in the laboratory database and a report is sent to the veterinary officer at the port of entry indicating the status of *Salmonella* in the product [39, 40].

### Data source and management

To achieve the objective of this study, laboratory records containing *Salmonella* results of MRPM imported into South Africa through the Durban port of entry from May 2016 to December 2017 were used. The following fields were extracted from the database country of origin, *Salmonella* results, sampling date, and importer. The dataset was assessed for missing values and incomplete information. None were observed in this dataset.

In total, 16 countries exported MRPM through the selected port of entry. However, 14 countries had exported <1% of consignments and were re-categorized as “All others.” Similarly, importers who imported <1% of consignments through the selected port of entry were re-categorized as “All others.” The variable “Season” was categorized into summer (November–March), autumn (April–May), winter (June–August), and spring (September–October).

### Statistical analysis

Statistical analyses were performed using the SAS 9.4 (SAS Institute Inc., Cary, NC, USA) statistical package. Crude and factor-specific proportions of *Salmonella* positive MRPM and their 95% confidence intervals were calculated. Associations among categorical variables, country of origin, month, season, year, importer, and MRPM *Salmonella* status, were assessed using the Chi-square or Fisher’s Exact tests where appropriate. The significance level was set at α = 0.05 for all the statistical tests.

### Predictors of *Salmonella* infection

The predictors of MRPM consignment that tested positive for *Salmonella* were assessed using a logistic regression model. First, a univariable logistic regression model was used to assess the association among exploratory variables, country, season, importer, year, and MRPM *Salmonella* status. The predictors with p < 0.20 were considered for inclusion in the multivariable logistic regression model.

Second, a multivariable logistic regression model using backward selection was fitted to the data containing all categorical variables with potential association (p ≤ 0.2) with the outcome. The significance of the predictor variables was set at a = 0.05. Confounding was assessed by comparing the change in model coefficients with and without the suspected confounders. If removing a suspected confounding variable resulted in a 20% or greater change in another model coefficient, the removed variable was considered a confounder and retained in the model regardless of its statistical significance. Adjusted odds ratios and their 95% confidence intervals were calculated for all four predictors retained in the final model. The Hosmer-Lemeshow goodness of fit test was used to assess the goodness of fit of the final model.

## Results

### The proportion of *Salmonella* positive consignments

In total, 8127 consignments were imported, of these, 5733 (70.54%) were tested for *Salmonella* species and 985 (17.18%, 95% Confidence Interval: 16.23–18.18) tested positive for *Salmonella* species. Most *Salmonella* positive consignments came from Country-B (26.95%) followed by Country-A (16.89%). Importer-VI (26.10%) had the highest proportion of positive consignments followed by importer-IV (18.80%). The year 2016 (21.12%) had more consignments testing positive for *Salmonella* species compared to the year 2017 (14.45%). The proportions of consignments testing positive for *Salmonella* also differed by season (Table-1).

**Table-1:** Proportion of *Salmonella* positive consignments imported through a port of entry in South Africa from May 2016 to December 2017.

Variable	Consignments tested			*Salmonella* positive		
	
	n^a^	%	95% CI^b^	n^a^	%	95% CI^b^
Country						
Country-A	5125	89.39	88.57–90.17	866	16.89	19.15–21.57
Country-B	319	5.56	5–6.18	86	26.95	30.97–43.27
All others	289	5.04	4.50–5.63	33	11.41	9.32–17.55
Importer						
I	2285	40.12	38.86–41.41	365	15.97	14.53–17.53
II	828	14.54	13.65–15.48	97	11.70	9.69–14.08
III	559	9.81	9.07–10.62	73	13.05	10.52–16.11
IV	449	7.88	8.05–9.52	79	18.80	12.89–19.29
V	440	7.72	7.06–8.45	42	9.54	7.14–12.65
VI	295	5.18	4.63–5.78	77	26.10	21.42–31.4
VII	220	3.86	3.39–4.39	31	14.09	10.11–19.31
All others	352	6.04	5.42–6.64	99	28.12	23.68–33.04
Month						
January	280	4.77	4.25–5.35	20	7.14	4.67–10.77
February	283	4.83	4.30–5.40	29	10.24	7.23–14.33
March	427	7.28	6.64–7.97	68	15.92	12.76–19.7
April	257	4.38	3.88–4.93	54	21.01	16.48–26.42
May	223	3.80	3.34–4.32	45	20.17	15.44–25.93
June	489	8.34	7.65–9.07	54	11.04	8.56–14.13
July	591	10.08	9.33–10.87	104	17.59	14.74–20.87
August	787	13.42	12.57–14.31	162	20.58	17.91–23.55
September	910	15.52	14.61–16.46	174	19.12	16.7–21.8
October	687	11.71	10.92–12.56	101	14.70	12.25–17.55
November	518	8.83	8.13–9.58	136	26.25	22.65–30.21
December	413	7.04	6.41–7.72	84	20.33	16.74–24.49
Year						
2016	2746	46.82	45.55–48.1	580	21.12	19.64–22.69
2017	3119	53.18	51.9–54.45	451	14.45	13.27–15.74
Season						
Autumn	480	8.18	7.50–8.91	99	20.62	17.25–24.47
Spring	1597	27.23	26.11–28.38	275	17.21	15.45–19.15
Summer	1921	32.75	31.56–33.97	337	17.54	15.91–19.31
Winter	1867	31.83	30.65–33.04	320	17.13	15.5–18.92

a Number,

b Confidence interval

**Figure-1:**
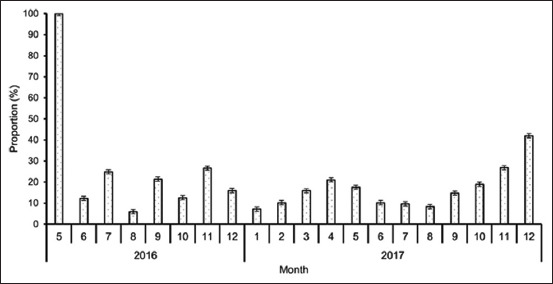
Monthly distribution in the proportion of consignments positive for *Salmonella* species from May 2016 to December 2017.

Over the study period, there were monthly variations in the proportions of consignments testing positive for *Salmonella* (Figure-1). In addition, there was a slight increase (p < 0.0001, Adjusted R^2^=0.0039) in the monthly proportions of *Salmonella* positive consignments.

### Description of *S. enterica* serotypes

Only 364 *Salmonella* isolates were serotyped in this study. Of these, *Salmonella* Heidelberg (73.90%) was the most common, followed by *Salmonella* Infantis (6.50%), *Salmonella* Salamae (4.67%), and *Salmonella* Schwarzengrund (3.57%) (Table-2).

**Table-2:** Distribution of *Salmonella enterica* serotypes reported from consignments imported in South Africa from May 2016 to December 2017.

Serotype	Frequency	Percentage
*Salmonella* Heidelberg	269	73.90
*Salmonella* Infantis	24	6.59
*Salmonella* Salamae	17	4.67
*Salmonella* Schwarzengrund	13	3.57
*Salmonella* Enteritidis	6	1.65
*Salmonella* Minnesota	6	1.65
*Salmonella* Muenchen	5	1.37
*Salmonella* Rough	5	1.37
*Salmonella* Coromandel	4	1.10
*Salmonella* Saintpaul	3	0.82
*Salmonella* Virginia	3	0.82
*Salmonella* Abony	1	0.27
*Salmonella* Brancaster	1	0.27
*Salmonella* Derby	1	0.27
*Salmonella* Fillmore	1	0.27
*Salmonella* Gloucester	1	0.27
*Salmonella* Irumu	1	0.27
*Salmonella* Tshiongwe	1	0.27
*Salmonella* Typhimurium	1	0.27
*Salmonella* Worthington	1	0.27

### Predictors of *Salmonella* species among the consignments

Based on the univariable logistic regression model, country (p < 0.0001), importer (p = 0.0001), season (p < 0.0001), and year (p = 0.0001) were potential predictors of a consignment testing positive for *Salmonella* species at α ≤ 0.20. Therefore, they were included in the multivariable model (Table-3).

**Table-3:** Univariable and multivariable logistic models showing predictors of *Salmonella* among MRPM consignments imported from May 2016 to December 2017.

Variable	Univariable logistic regression				Multivariable logistic regression			
	
	OR^a^	95% CI^b^		p-value	OR^a^	95% CI^b^		p-value
Country				<0.0001				
A	1.577	1.090	2.283		1.879	1.255	2.812	0.6743
B	2.863	1.846	4.441		3.958	2.421	6.470	<0.0001
All others	Ref	-	-		Ref	-	-	-
Season				<0.0001				
Autumn	1.256	0.977	1.616		1.488	1.124	1.969	<0.0001
Spring	1.006	0.842	1.200		0.767	0.629	0.935	0.0004
Summer	1.029	0.869	1.217		0.843	0.698	1.018	<0.0001
Winter	Ref	-	-		Ref	-	-	-
Importer				0.0001				
VI	1.476	1.056	2.064		1.110	0.933	0.792	<0.0001
II	0.555	0.412	0.749		0.471	0.350	0.634	0.0706
I	0.420	0.285	0.619		0.657	0.519	0.832	0.0553
IV	0.768	0.604	0.977		0.461	0.323	0.658	0.1114
III	0.531	0.330	0.853		0.544	0.394	0.751	0.6792
VII	0.637	0.454	0.893		0.558	0.360	0.864	0.8862
V	0.593	0.427	0.825		0.238	0.160	0.355	<0.0001
All others	Ref	-	-		Ref	-	-	-
Year				<0.0001				
2016	1.584	1.383	1.814		1.677	1.414	1.989	<0.0001
2017	Ref	-	-		Ref	-	-	-

a Odds Ratio,

b Confidence interval

In the final model, the odds of a consignment testing positive for *Salmonella* spp. was significantly higher among those from Country-B (Odds Ratio [OR]: 3.958, p < 0.0001) compared to “All others.” Consignments imported in 2016 were 1.563 times (p < 0.0001) as likely to test positive for *Salmonella* when compared to those imported in 2017. Seasonally, the odds of an imported consignment testing positive for *Salmonella* was higher in autumn (OR: 1.488, p < 0.0001) but lower in summer (OR: 0.843, p < 0.0001) and spring (OR: 0.767, p = 0.0004) when compared to the winter season. Importer-V had a lower odds (OR: 0.238, <0.0001) of importing a consignment positive for *Salmonella* species compared to “All others.” While the odds of importing a *Salmonella* positive consignment was higher among consignments imported by Importer-VI (OR: 0.461, <0.0001) when compared to “All others” (Table-3).

## Discussion

Salmonellosis remains a major public health concern globally [20]; thus, research on MRPM as a potential source of *Salmonella* species is important in guiding risk mitigation strategies for the global spread of salmonellosis. Moreover, 600 million diarrheal cases globally have been associated with non-typhoidal *Salmonella* spp. [42]. In addition, non-typhoidal *Salmonella* spp. significantly contributes to high incidences of foodborne illnesses in South Africa [43, 44].

In this study, the proportion of *Salmonella* positive MRPM consignments was higher than the 15% that was reported in California [45] and 13% reported in Belgium [46]. In contrast, higher prevalences of *Salmonella* species in MRPM have been reported in the United States (44.6%) [47] and Estonia (38.5%) [48], while Rania and Qasem reported no *Salmonella* species from MRPM in Jordan [49]. The results of this study suggest the risk of importing MRPM contaminated with *Salmonella* species exists [50]. Therefore, a quantitative risk assessment must be undertaken to evaluate the effectiveness of current mitigation measures and make recommendations for improvement where necessary. This step is important as South Africa imports almost all of its MRPM from other countries [51]. Without, proper risk assessment, a breakdown in the biosecurity measures may lead to future outbreaks of salmonellosis in South Africa [2, 5, 47, 52].

The majority of *Salmonella* serotypes reported in this study were from *enterica* species which is known to be zoonotic and is of global public health concern [53, 54]. For example, *Salmonella* Heidelberg identified in this study was reported in 96% of inmates who consumed MRPM in the United States [55]. Similarly, *Salmonella* Schwarzengrund, *Salmonella* Infantis [56], and *Salmonella* Enteritidis [57] reported in this study were also reported in other studies globally. The presence of these zoonotic pathogens requires that the current South African standard operating procedure (SOP) allows for the importation of *Salmonella* positive meat commodities except for *Salmonella* Typhi, *Salmonella* Enteritidis, and *Salmonella* Typhimurium serotypes and where no more than two out of five samples tested positive for *Salmonella* species be reviewed [58]. The SOP could be aligned with the South African regulations of Meat Safety Act 40 of 2000 on poultry and poultry products which require that biological hazards be identified, prevented, eliminated, or reduced to an acceptable level [59, 60].

In the European Union, rejections of poultry products are mostly associated with *Salmonella* contamination [61]. The Commission Regulation (EC) No 2073/2005 of 15 states that MRPM consignments must have zero samples testing positive for *Salmonella* species [62, 63]. While in the US, the Food Safety and Inspection Service accepts no more than five positive *Salmonella* carcasses out of 51 samples [64]. These countries make up the biggest share of poultry meat and poultry products imported into South Africa [65]. Therefore, aligning the South African regulatory framework to the international best practice will help reduce the risk of importing *Salmonella*-contaminated MRPM consignment to negligible levels [58].

The odds of MRPM consignment testing positive for a *Salmonella* species significantly differed based on the country of origin. This is not surprising as the country of origin and the area within the country have been shown to influence the occurrence of *Salmonella* in poultry and poultry products [66–68]. This could be attributed to differences in the legislation, hygiene practices, biosecurity, disease control, and farming practices [40–41]. Therefore, when assessing the risk of importing *Salmonella-*contaminated consignments into South Africa, the prevalence of the pathogen in poultry and poultry products at country and region level must be taken into consideration [69].

Mechanically recovered poultry meat consignment imported in 2016 when compared to 2017 had an increased odds of testing positive for *Salmonella*. The reason for this difference is unclear in the literature. However, this could be due to changes in the enforcement protocols at the port of entry and improved process control in the exporting countries [47]. In addition, there was seasonality in the proportion of MRPM consignments that tested positive for *Salmonella* species. Other studies have also reported seasonality in the proportion of poultry products testing positive for *Salmonella* species [70, 71]. Therefore, the risk-adjusted model for the importation of *Salmonella* positive MRPM consignment must also consider the yearly and seasonal variation of disease occurrence.

An importer was also a significant predictor of the likelihood of a consignment testing positive for *Salmonella* species. This is to be expected since importers are the ones who decide on the preferred country of importation. In view of this, importers should form part of the risk management strategy to ensure that they import consignments from locations where the risk is negligible.

## Limitations of the study

The authors did not have control over the variables and the data collection process. In addition, the study focuses on the MRPM imported through a single port of entry. Therefore, the results of this study may not reflect situations at all ports of entry in South Africa. In view of this, more studies are needed to investigate *Salmonella* species in MRPM consignments imported through other ports of entry into South Africa. Nonetheless, the findings of this study contribute significantly to our understanding of the risk of importing MRPM contaminated with *Salmonella* species into South Africa.

## Conclusion

*Salmonella* species were isolated from MRPM consignment imported into South Africa over the study period. *Salmonella* positive consignments differed based on the country of origin, the importer, and the time of importation. Since *Salmonella* species associated with foodborne illness were reported in this study, the procedure for handling *Salmonella*-contaminated MRPM must be amended in line with Meat Safety Act, 2000 (Act No. 40 of 2000) and the international best practice. Furthermore, a quantitative risk assessment must be undertaken to quantify the likelihood of importing a *Salmonella*-contaminated MRPM consignment. Furthermore, the risk assessment can be used to identify potential drivers for contamination throughout the value chain.

## Authors’ Contributions

TNN, KM, and DNQ: Study design. KM and DNQ: Drafting of manuscript and data analysis. KM: Extensive review of the manuscript. All authors have read, reviewed, and approved the final manuscript.
